# The association of NICU capacity strain with neonatal mortality and morbidity

**DOI:** 10.1038/s41372-025-02449-0

**Published:** 2025-10-20

**Authors:** Elizabeth G. Salazar, Molly Passarella, Brielle Formanowski, Jeannette Rogowski, Erika M. Edwards, Scott D. Halpern, Ciaran Phibbs, Scott A. Lorch

**Affiliations:** 1 Division of Neonatology, The Children’s Hospital of Philadelphia, Philadelphia, PA USA; 2 Perelman School of Medicine at the University of Pennsylvania, Philadelphia, PA USA; 3 Leonard Davis Institute of Health Economics, Philadelphia, PA USA; 4 The Pennsylvania State University, University Park, PA USA; 5 Vermont Oxford Network, Burlington, VT USA; 6 Department of Pediatrics, The Robert Larner MD, College of Medicine, The University of Vermont, Burlington, VT USA; 7 Department of Mathematics and Statistics, The University of Vermont, Burlington, VT USA; 8 Division of Critical Care Medicine, The University of Pennsylvania, Philadelphia, PA USA; 9 Stanford University School of Medicine, Stanford, CA USA; 10 Veterans Affairs Palo Alto Health Care System, Palo Alto, CA USA

**Keywords:** Paediatrics, Health services

## Abstract

**Objective:**

To examine the association of admission NICU capacity strain with neonatal mortality and morbidity.

**Study design:**

2008–2021 South Carolina cohort using linked vital statistics and discharge data of 22–44 weeks GA infants, born at hospitals with ≥ level 2 unit and ≥5 births <34 weeks GA/year. The exposure was deciles of admission capacity strain, defined as the sum of infants ≤44 weeks GA with a congenital anomaly plus infants <34 weeks GA. The primary outcome was a composite of mortality and term and preterm complications. We used Poisson generalized linear mixed models to examine the association of exposure with outcome adjusting for patient and hospital characteristics.

**Results:**

We studied 64,647 infants from 30 hospitals. High capacity strain was associated with increased risk of mortality and morbidity adjusting for patient/hospital factors (for example, tenth decile aIRR 1.14, 95% CI 1.03–1.27).

**Conclusion:**

Capacity strain is associated with adverse NICU outcomes.

## Introduction

Intensive care unit (ICU) capacity strain is the ICU’s time-varying stressors, including demand from patient census and acuity as well as current resource availability [1]. Capacity strain is influenced by physical and human resources as well as patient volumes, acuity, and specialized needs [2]. Measures of ICU capacity strain frequently encompass the concepts of patient census, acuity, admissions, discharges, and staffing. The most commonly used measures of adult ICU capacity strain include ICU census (often risk adjusted) and number of admissions [3, 4]. Increased ICU capacity strain on an adult patient’s day of admission is associated with decreased in-hospital survival and safety practices [3, 5–8]. Despite strong evidence in the adult ICU, the role of neonatal intensive care unit (NICU) capacity strain as a driver of hospital variation has been understudied.

**Fig. 1:**
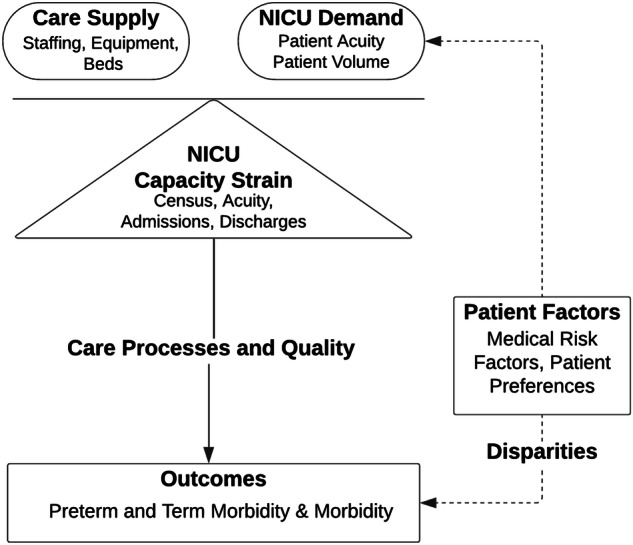
NICU Capacity Strain Conceptual Model. The relationship between NICU capacity strain and its measures is seen with care supply and demand, as well as its impact on patient care processes, quality, and outcomes.

Annually in the United States, there is substantial variability in NICU outcomes, including 15-fold variation in hospital preterm mortality rates and 1- to-3-fold variation in complication rates [9–12]. Persistent hospital outcome variation while controlling for patient acuity suggests that hospital factors play a role in this variation [12, 13]. While available hospital resources, measured through American Academy of Pediatrics neonatal levels of care [14], and experience with the neonatal patient population, measured by volume [15], are both associated with neonatal outcomes, little research has examined the role of NICU capacity strain on neonatal outcomes, despite a robust association between ICU capacity strain and adult outcomes [15–17]. Limited published studies report an association of NICU census with risk of infection in very preterm infants [18] and census with likelihood of discharge [19]. By influencing quality of care delivered, NICU capacity strain is hypothesized to ultimately influence patient outcomes (Conceptual Model, Fig. 1). Recent workforce shortages in pediatrics, both physician and nurse, make understanding the role of ICU capacity strain particularly critical [20].

The study objective was to examine the association of NICU capacity strain at admission with in-hospital term and preterm mortality and complication. We chose to focus on capacity strain at admission as prior research supports both that delivery hospital has a strong influence on neonatal outcomes [13] and admission ICU capacity strain, measured by standardized acuity-adjusted census, is associated with adult mortality [3]. We used a novel measure of NICU capacity strain that incorporates the census of high-risk infants by measuring the standardized daily sum of infants <44 weeks gestational age (GA) with a congenital anomaly plus all infants born <34 weeks GA, and also examined the role of admissions. We hypothesized that increased NICU capacity strain would be associated with worse neonatal outcomes when controlling for both patient-level and hospital-level factors.

## Methods

### Data and study population

Using 2008 to 2021 linked South Carolina vital statistics and hospital records, we conducted a retrospective cohort study of infants born between 22 and 44 weeks GA. Infant birth and death certificate data were linked to birthing parent and infant hospital administrative data by the South Carolina Department of Health with a reported birthing parent-infant match rate of 96–99% using described methods [21]. We used the merged American Hospital Association (AHA) Annual Survey of Hospitals data to obtain hospital characteristics [22]. To identify infants that were cared for in the NICU, included infants were born at hospitals with neonatal care level 2 or greater and with ≥5 births of infants <34 weeks GA per year and received a revenue code of level 2 or greater, which is consistent with the receipt of NICU care (Supplementary Fig. 1). Infants were also excluded if they had missing birth certificate, birthing parent or infant hospital data or a birth weight greater than five standard deviations from the mean for GA, suggesting that one or both of these variables were miscoded [16]. The study cohort was 11% of the entire birth cohort, consistent with prior estimations of NICU admissions at 12% [9].

This study followed the Strengthening the Reporting of Observational Studies in Epidemiology (STROBE) guidelines [23]. The Children’s Hospital of Philadelphia institutional review board determined that the abstracted data did not meet the requirements of human subjects research. Data use was approved by the South Carolina Department of Health.

### Study measures and variables

The primary exposure of interest was the NICU capacity strain at admission. Admission NICU capacity strain was defined using a standardized measure of the daily census of higher-risk infants, defined as infants ≤44 weeks GA with a congenital anomaly plus all infants born <34 weeks GA. To define NICU capacity strain, we defined high-risk infants as those ≤44 weeks GA with a congenital anomaly as we know these infants are more likely to require resource-intensive care, and thus serve as a proxy for unit-level acuity [24]. We did not include other term infants in our definition of high-acuity infants as the majority of term infants were likely to be low-acuity, and we lacked necessary clinical data, such as daily ventilation to identify other high-acuity-term infants. We also defined all infants born <34 weeks GA as high-risk because these infants would all routinely be cared for in the NICU and be at risk for increased resource utilization and potential acuity [25]. Standardization was performed by subtracting the average annual hospital census of this higher-risk population from the daily census of this higher-risk population and then dividing by the average daily hospital census of this higher-risk population for that given year. Standardizing by a unit’s annual average daily census of high-risk infants distinguishes this value from volume, which does not incorporate aspects of acuity and is not standardized. After examining the functional form of capacity strain, NICU capacity strain was divided into deciles with a separate category for patients born on days of zero capacity strain. The second capacity strain decile was chosen as a referent to allow for both examination of higher levels of capacity strain and the zero and low capacity strain groups. Capacity strain was assigned based on the value for the day prior to the patient’s birth to ensure the exposure occurred prior to the outcome. We chose a definition of NICU capacity strain that intentionally did not incorporate care resources, such as nursing staffing ratios, as these data are typically unavailable for hospitals on a daily basis and annual values vary by other hospital factors such as financial health and stability that could confound any relationship observed in the study [26]. We also examined the total number of NICU admissions on a given day as an alternate definition of NICU capacity strain in our sensitivity analyses, as this is another well-studied measure of adult ICU capacity strain [3]. As the concept of capacity strain expands beyond that of volume alone, acknowledging the role that acuity plays in increasing the demand for care in addition to patient volume, we did not examine standardized patient volume alone.

The primary outcome was a composite of mortality and complications for all term and preterm infants. For preterm infants, complications included severe (grade 3 or 4) intraventricular hemorrhage, necrotizing enterocolitis, surgical retinopathy of prematurity, chronic lung disease or infection as defined by ICD codes (Supplementary Table 1) [27]. For term infants, complications included moderate and severe unexpected newborn complications as previously defined [28]. Outcomes for infants transferred after delivery were assigned to the birth hospital given literature supporting the role of the delivery hospital on neonatal outcomes [13].

### Covariate definitions

Patient-level covariates included gestational age (by week), infant sex [29], small for gestational age (<10th percentile for GA using Fenton growth chart) [30, 31], multiple gestation [29], and congenital anomaly [24], which have all been previously associated with the outcome measures. Birthing parent covariates included race and ethnicity [32], age [33], insurance [34], education [35], smoking [36], any diabetes [37], any hypertension [38], BMI (body mass index) [39], mode of delivery [40], and birth year [41] given known association with outcomes. We also examined hospital characteristics including ownership [42], rurality [42], number of NICU beds [43], NICU level of care [44], and annual birth volume [15] (Supplementary Table 2).

### Statistical analyses

**Fig. 2:**
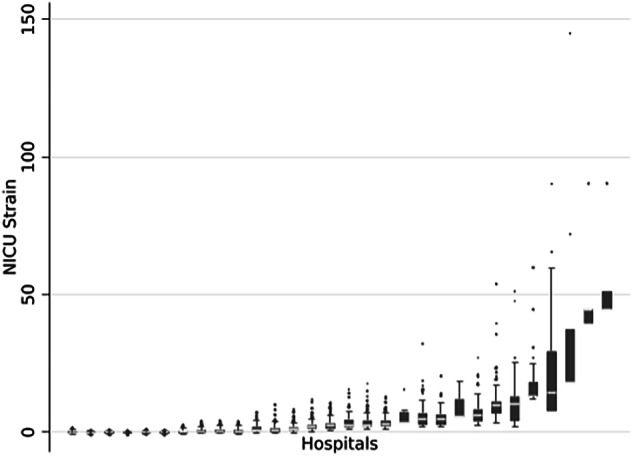
Variation in NICU capacity strain by hospital. NICU capacity strain was calculated as a standardized measure of the daily census of infants <44 weeks GA with a congenital anomaly plus infants born <33 weeks GA. Standardization was performed by calculating this daily census minus the average annual hospital census, divided by the average annual hospital census. GA gestational age, NICU neonatal intensive care unit.

**Fig. 3:**
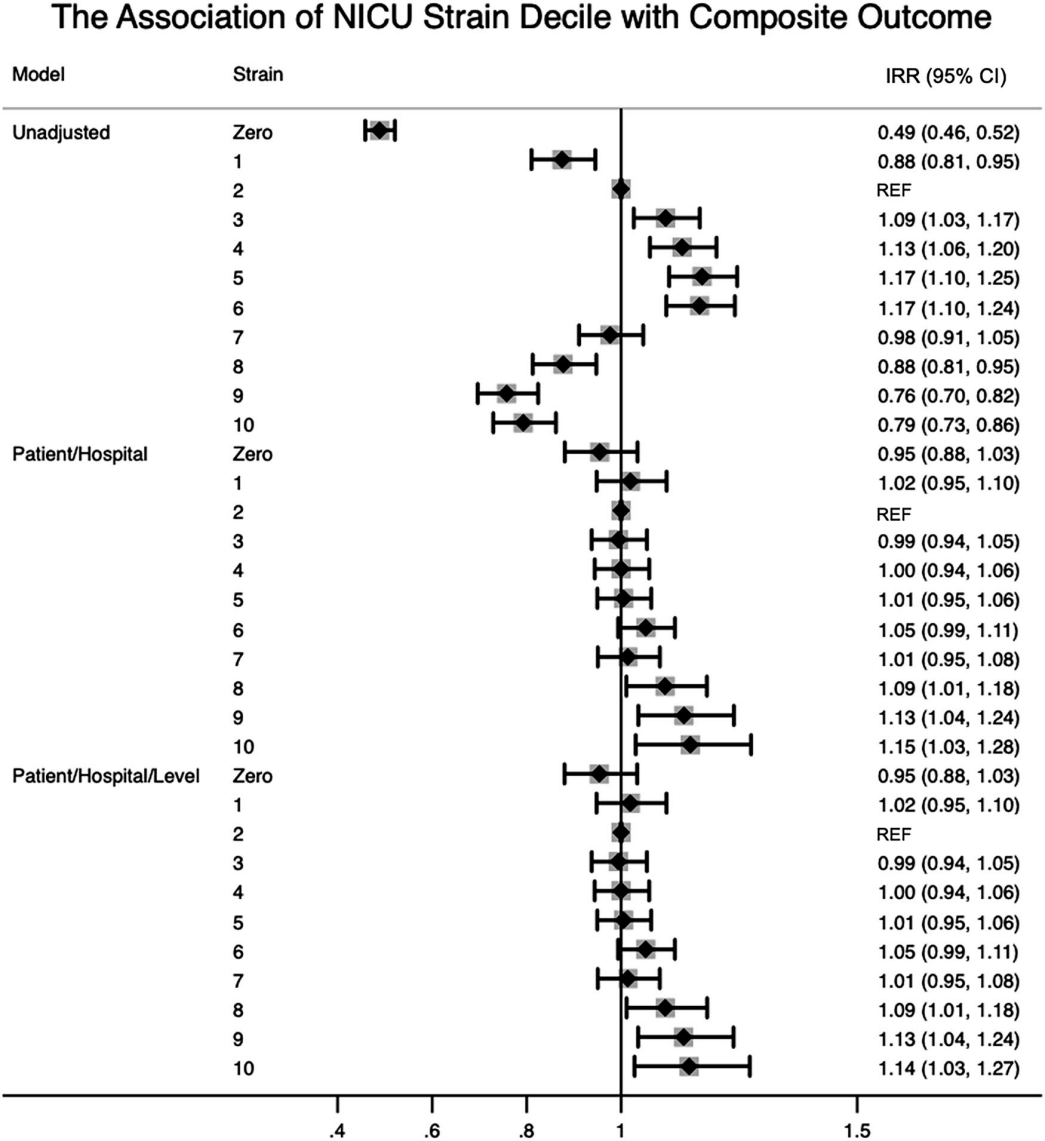
Association of NICU capacity strain with composite primary outcome in unadjusted and adjusted models. Models were multivariable modified poisson generalized linear mixed models. Patient characteristics include birthing parent variables (age, race and/or ethnicity, diabetes, hypertension, BMI, smoking, insurance, education, and cesarean section) and infant variables (gestational age, gender, small for gestational age, multiple gestation, multiple congenital anomalies). Hospital was controlled for with a fixed effect. AAP NICU level was included for the final set of models. Birth year was included in all models.

**Fig. 4:**
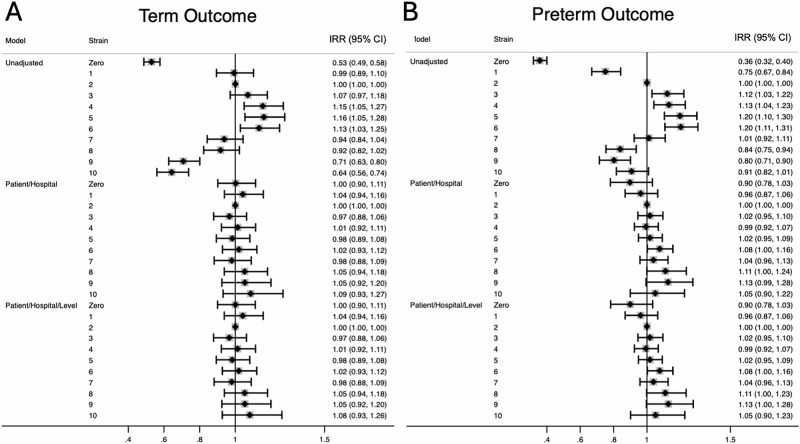
Association of NICU capacity strain with preterm and term mortality and morbidity in unadjusted and adjusted models. Panel **A** indicates models for term outcomes while Panel **B** indicates models for preterm outcomes. Models were multivariable modified Poisson generalized linear mixed models. Patient characteristics include birthing parent variables (age, race and/or ethnicity, diabetes, hypertension, BMI, smoking, insurance, education, and cesarean section) and infant variables (gestational age, gender, small for gestational age, multiple gestation, multiple congenital anomalies). Hospital was controlled for with a fixed effect. AAP NICU level was included for the final set of models. Birth year was included in all models.

Descriptive statistics were reported using counts and percentages for categorical variables and means and SDs for continuous variables. We evaluated associations using χ2 tests for dichotomous variables and analysis of variance for continuous variables. We examined the hospital-level variability of patient-level NICU capacity strain using boxplots (Fig. 2). We examined the overall association of deciles of NICU capacity strain with mortality and complication by using multivariable Poisson generalized linear mixed models with adjustment for birthing parent and infant to estimate risk ratios (Figs. 3 and 4). We included a fixed effect for hospital in the model, which accounts for unchanging factors within a given hospital over the time period of the study. Inclusion of this hospital fixed effect allows for one to interpret the capacity strain variable in these models as the effect of changes in capacity strain within a given hospital on outcomes. Without such a variable, our analyses may be biased by unobserved hospital factors more likely to occur within hospitals of with larger capacity strain, similar to the bias observed in hospitals of higher level or volume. We also used a fixed effect for hospital with neonatal level of care to isolate the effect of level of care while also addressing other hospital specific variation. Analyses were performed using Stata, version 18.

### Sensitivity analyses

We performed several sensitivity analyses to ensure the robustness of our findings. We examined the functional form of capacity strain, performing analyses with it as a continuous variable and in quintiles, with consistent results. We also employed an alternate measure of standardization, dividing by the 95% value of annual hospital census, with consistent results. Next, we examined the number of NICU admissions as an alternative measure of NICU capacity strain and did not find an association with overall, term, or preterm outcomes (Supplementary Figs. 2 and 3). Finally, we also excluded level 2 units and patients with zero values for capacity strain and found consistent results (Supplementary Fig. 4 and 5).

## Results

The study cohort included 64,647 infants from 30 hospitals (273 hospital-years). There were 29,605 term (46%) and 35,042 preterm (54%) infants. In the cohort, 15,700 infants (24%) experienced zero capacity strain; 14,409 (23%) experienced low capacity strain (deciles 1–3); 23,941 (37%) experienced typical capacity strain (deciles 4–7); and 10,597 (16%) experienced high capacity strain (deciles 8–10) at admission (Table 1). Infants exposed to typical capacity strain were more frequently preterm, multiple gestation, had a major congenital anomaly, and were born to Black birthing parents with government insurance, diabetes, hypertension, and obesity and delivered via cesarean section (Table 1). Infants born during a period of high capacity strain (deciles 4–7) were also more likely to be born at lower level NICUs with lower annual birth volume (Supplementary Tables 2, 3).

**Table 1:** Perinatal characteristics overall and by NICU capacity strain categories.

Characteristics	Overall	Zero capacity strain	Low capacity strain (Deciles 1–3)	Typical capacity strain (Deciles 4–7)	High capacity strain Deciles (8–10)
	*N* (%)	*N* (%)	*N* (%)	*N* (%)	*N* (%)
Patient	*N* = 64,647	*N* = 15,700	*N* = 14,409	*N* = 23,941	*N* = 10,597
Female sex^a^	29,538 (46)	7043 (44)	6524 (45)	11,125 (47)	4846 (46)
Gestational age subgroup^b^					
<28 weeks GA	3433 (5)	0 (0)	940 (7)	2042 (9)	451 (4)
28–31 weeks GA	6014 (9)	0 (0)	1445 (10)	3765 (16)	804 (8)
32–34 weeks GA	14,323 (22)	1461 (9)	3410 (24)	6631 (28)	2821 (27)
35–36 weeks GA	11,272 (17)	3561 (23)	2503 (17)	3416 (14)	1792 (17)
>37 weeks GA	29,605 (46)	10,678 (68)	6111 (42)	8087 (34)	4729 (45)
Small for gestational age^b^	9611 (15)	2290 (15)	2300 (16)	3658 (1)	1363 (13)
Multiple gestation^b^	8730 (14)	1174 (7)	1821 (13)	4183 (18)	1552 (15)
Major congenital anomaly^b^	4218 (7)	38 (0.2)	1037 (7)	2351 (10)	792 (8)
Birthing parent					
Race and ethnicity^b^					
Asian/ Pacific Islander	668 (1)	182 (1)	145 (1)	237 (1)	104 (1)
Black	23,911 (37)	4922 (31)	5589 (39)	9633 (40)	3767 (36)
Hispanic	2335 (4)	432 (3)	588 (4)	1023 (4)	292 (3)
Other	3209 (5)	761 (5)	728 (5)	1191 (5)	529 (5)
White	34,524 (53)	9403 (60)	7359 (51)	11,857 (50)	5905 (56)
Age (years)^b^					
<20	5304 (8)	1348 (9)	1193 (8)	1913 (8)	850 (8)
20–24	16,514 (26)	4207 (27)	3664 (25)	5790 (24)	2853 (27)
25–34	33,420 (52)	8049 (51)	7422 (52)	12,556 (53)	5393 (51)
35–39	7533 (12)	1718 (11)	1694 (12)	2915 (12)	1206 (11)
≥40	1876 (3)	378 (2)	436 (3)	767 (3)	295 (3)
Insurance^b^					
Government	37,647 (58)	8860 (56)	8518 (59)	14,280 (60)	5989 (57)
Private	25,335 (39)	6554 (42)	5478 (38)	8912 (37)	4391 (41)
Self-Pay	1364 (2)	248 (2)	339 (2)	593 (3)	184 (2)
Other	301 (0.5)	38 (0.2)	74 (1)	156 (1)	33 (0.3)
Education (years)					
8th grade or less	1502 (2)	318 (2)	333 (2)	632 (3)	219 (2)
9–12th grade, no degree	9410 (15)	2270 (14)	2043 (14)	3627 (15)	1470 (14)
High School/GED	16,718 (26)	4104 (26)	3806 (26)	6138 (26)	2670 (25)
Some college	22,752 (35.)	5465 (35)	5103 (35)	8306 (35)	3878 (37)
4-year college	8976 (14)	2236 (14)	1965 (14)	3276 (14)	1499 (14)
>4-year college	5062 (8)	1237 (8)	1117 (8)	1899 (8)	809 (8)
Missing	227 (0.4)	70 (0.5)	42 (0.3)	63 (0.3)	52 (0.5)
Any smoking during pregnancy	13,000 (20)	3131 (20)	2829 (20)	4907 (21)	2133 (20)
Diabetes^b^	9199 (14)	2059 (13)	2159 (145)	3747 (16)	1234 (12)
Hypertension^b^	10,672 (17)	2014 (13)	2655 (18)	4694 (20)	1309 (12)
BMI^b^					
Underweight	2447 (4)	601 (4)	560 (4)	861 (4)	425 (4)
Normal	22,627 (35)	5685 (36)	4881 (34)	8314 (35)	3747 (35)
Overweight	15,227 (24)	3668 (23)	3413 (24)	5619 (24)	2527 (24)
Obese	16,880 (26)	3950 (25)	3893 (27)	6348 (27)	2689 (25)
Obese+	6130 (9)	1243 (8)	1487 (10)	2518 (11)	882 (8)
Missing	1336 (2)	553 (4)	175 (1)	281 (1)	327 (3)
Cesarean^b^	35,326 (55)	7674 (49)	7940 (55)	14,153 (59)	5559 (53)
Composite outcome^b^	14,633 (23)	1950 (12)	3683 (26)	6813 (29)	2187 (21)
Term outcome^b^	6528 (22)	1512 (14)	1669 (27)	2379 (29)	968 (21)
Preterm outcome^b^	8105 (23)	438 (9)	2014 (24)	4434 (28)	1219 (21)

Please see methods for descriptions of covariates and outcomes.

*GA* gestational age, *GED* general education development, *NICU* neonatal intensive care unit.

^a^Indicates *p* < 0.05.

^b^Indicates *p* < 0.01.

Figure 2 depicts the within and between variability of admission NICU capacity strain by hospital. Hospitals with average daily hospital census of higher risk infants less than 1 had larger values of NICU capacity strain, as the NICU capacity strain measure was created by dividing by a number less than 1. Including a hospital fixed effect in our models allows us to interpret the capacity strain variable as change of capacity strain within a given hospital, facilitating interpretation of these findings in the midst of this hospital-level variability.

In unadjusted bivariate analyses, infants born during times of typical capacity strain (deciles 4–7) more frequently experienced composite, term, and preterm adverse outcomes (29% in the typical capacity strain vs. 21% and 26% in high and low capacity strain, respectively; Table 1 and Fig. 3). In unadjusted analyses, there was a decreased incidence rate ratio of the primary outcome in infants exposed to the highest capacity strain deciles (tenth decile IRR 0.79, 95% CI 0.73–0.86) and lowest capacity strain deciles (first decile IRR 0.88, 95% CI 0.81–0.95), compared to the second capacity strain decile.

In models adjusted for patient covariates with a fixed effect for hospital, birth during periods of high NICU capacity strain in a given hospital was associated with an increased risk of mortality and complication compared to birth during the second capacity strain decile. This suggests that within a single hospital, birth during periods of increased NICU capacity strain was associated with increased mortality and complication when controlling for patient covariates. When adjusting for patient covariates with a fixed effect for hospital and neonatal level of care, birth during the highest deciles of capacity strain remained associated with increased mortality and complication (for example, the tenth decile aIRR 1.14, 95% CI 1.03–1.27, compared to the second capacity strain decile). Neonatal levels of care did not have a significant impact on outcomes (level 3 aIRR 0.81, 95% CI 0.59–1.12) when adjusting for capacity strain.

In models examining secondary term outcomes unadjusted for covariates, the highest capacity strain decile was associated with a lower relative risk of term (IRR 0.64, 95% CI 0.56–0.76, relative to second decile) adverse outcomes (Fig. 4A). After adjustment, however, none of the deciles of NICU capacity strain were associated with adverse outcomes.

For preterm infants, the eighth and ninth deciles of capacity strain were associated with decreased risk of adverse preterm outcomes before adjustment (eighth decile IRR 0.84, 95% CI 0.75–0.94; ninth decile aIRR 0.80, 95% CI 0.71,0.90; Fig. 4B). However, after adjustment for patient covariates with a fixed effect for hospital and neonatal levels of care, these deciles were associated with increased risk (eighth decile aIRR 1.11, 95% CI 1.00–1.23; ninth decile aIRR 1.11 95% CI 1.00–1.28).

Sensitivity analyses excluding level 2 NICUs demonstrated a persistent association of the highest decile of NICU capacity strain with adverse composite outcomes in models adjusting for patient and hospital factors (tenth decile aIRR 1.19, 95% CI 1.05–1.35, Supplementary Fig. 2). Additional analyses excluding zero values of capacity strain demonstrated a persistent association of the highest deciles of NICU capacity strain with adverse composite outcomes in models adjusting for patient and hospital factors (tenth decile aIRR 1.14, 95% CI 1.01–1.29; Supplementary Fig. 3).

In sensitivity analyses examining an alternate definition of NICU capacity strain, number of daily admissions, higher deciles of NICU capacity strain were significantly associated with the composite, term, or preterm outcomes in unadjusted models. This effect was no longer seen when adjusting for patient factors with a fixed effect for hospital alone as well as hospital and level of care (Supplementary Figs. 4, 5).

## Discussion

This study examines the association of NICU capacity strain at admission with neonatal mortality and complication using an adapted measure of ICU capacity strain for the NICU, capturing the standardized daily census of high-acuity infants care for within that NICU for a given year. In adjusted models for patient and hospital characteristics including level of care, exposure to high NICU capacity strain on admission was associated with increased risk of complication and mortality.

Consistent with the adult ICU literature associating ICU capacity strain with mortality and quality of care, this study is the first to report the association with NICU capacity strain at admission with neonatal mortality and complication [3, 5–8]. Similar to the original adult ICU study, we found that our novel standardized census measure was associated with less mortality and complications in unadjusted analyses, but that this association was reversed with adjustment for patient acuity [3]. This finding may be explained by the observation that patients experiencing higher capacity strain tended to be cared for in NICUs with lower level of care (levels 2 and 3), lower delivery volumes, less acute patients, and thus a lower likelihood of having an adverse outcome. Without adjusting for these hospital and patient trends, high capacity strain appears to be associated with improved outcomes. This study’s ability to control for hospital effects through a fixed effect, level, and patient acuity highlight the true impact of increased ICU capacity strain within a given unit.

The consistency of the association of standardized census with adverse outcomes in the adult and neonatal ICU is especially interesting given the different patient populations of the two units. NICU patients have a longer average length of stay (~13 days) [45] compared to an adult ICU (~3 days), as these infants require time for appropriate development of feeding, breathing, and thermoregulation, and there is often no alternate stepdown unit for transfer [46]. This may lead to increased numbers of lower acuity patients contributing to a standardized census capacity strain measure over time. While this study supports that capacity strain surrounding NICU admission is essential in shaping neonatal outcomes, additional work is needed to better understand how the same infant may differentially contribute to NICU capacity strain over the course of their admission [13].

This study also supports that NICU capacity strain may have distinct impact on different patient populations, such as term and preterm infants. While high NICU capacity strain upon admission was associated with adverse preterm outcomes, the association did not persist for term infants. This may be because preterm infants are more vulnerable to adverse outcomes under stressed conditions, especially in the first day after delivery. The impact of NICU capacity strain on process measures in term infants, such as performance of harm reduction practices or adherence to evidence-based care practices, has not yet been studied in the neonatal literature [5].

While multiple measures of capacity strain including acuity-adjusted census and admission are associated with adult mortality, this study found that risk-adjusted census, and not admissions, were associated with increased neonatal mortality and complication [3, 5, 7, 8]. One potential hypothesis for this finding is that births, and thus NICU admissions, may occur at a more regular cadence and thus cause less stress to the NICU system compared to an unplanned critical care admission.

This study also found that NICU capacity strain was significantly associated with adverse patient outcomes within hospital. This highlights that NICU capacity strain is an important separate hospital measure to consider as an influence on patient outcomes. It remains unknown how NICU capacity strain functions in each level of care, as well as the relationship between annual patient volume and capacity strain, as hospitals with increased annual patient volume may have built more robust systems to facilitate NICU capacity strain.

These results also highlight the importance of mitigating NICU capacity strain. Strategies including increased bed supply as well as increasing staffing resources may be considered. Longitudinal studies demonstrate a 42% increase in NICU beds from 1991 to 2017 without a clear relationship to associated increased in newborn risk [47]. National studies may consider how this increase in NICU bed supply has influenced NICU capacity strain over time. Additionally, the observed rise in discretionary NICU admissions for larger and less premature infants seen in recent years may also lead to increased capacity strain in the NICU, potentially negatively impacting patient outcomes [48, 49]. In considering staffing, prior research suggests that nurse-staffing ratios are highly relevant to the very preterm infant population [50]. Further investigation into how resources such as nurse: patient ratios, acuity-based nursing staffing, physician full-time equivalents, as well as physician; patient ratios influence the relationship between NICU capacity strain and patient outcomes is warranted, particularly in light of changing staffing models.

This study has limitations. First, this study is the first to reports the association of NICU capacity strain with patient outcomes in a single state. Examination of additional states is necessary to ensure generalizability. Second, we adjusted for patient and birthing parent covariates, including presence of congenital anomalies and gestational age, to capture patient acuity. While our data source did not contain additional clinical variables, such as days on the ventilator, to further control for patient acuity, prior work suggests that gestational age may serve as an adequate indicator of increased demand associated with adverse outcomes [51]. Further work describing how capacity strain changes over time and longitudinally impacts patient outcomes are warranted. Additionally, our measure’s simplicity may facilitate applicability. Future work with data sources including clinical variables will allow for more accurate identification of a high-risk and acuity population contributing to NICU capacity strain.

Despite these limitations, this study has many strengths. It uses a large, multiyear dataset of linked birthing parent-infant data capturing a variety of hospital types to create a novel definition of NICU capacity strain. It is the first study to report an association between NICU capacity strain and neonatal complication and mortality. Additionally, it controls for other key hospital-level drivers of outcomes through a fixed effect and incorporation of neonatal levels of care. Our work indicates that NICU capacity strain is an important and separate driver of outcome variability. By providing a definition of NICU capacity strain, this work offers a first look into better understanding how NICU capacity strain influences neonatal outcome variation and associated disparities.

## Conclusion

Exposure to high NICU capacity strain at admission was associated with increased risk of neonatal mortality and complication when adjusting for infant, birthing parent, and hospital characteristics, including level of care. Further research is merited to characterize the influence of NICU capacity strain on care processes and quality of care, as well as the influence of changing NICU capacity strain over the course of the hospitalization. Future studies are needed to understand if available hospital resources, such as nurse to patient ratios, can mitigate the adverse effects of high NICU capacity strain. Ultimately, this work supports the importance of capacity management in the NICU to optimize patient outcomes.

## Supplementary information

Supplemental Material
