# Supplementary material for: The association of NICU capacity strain with neonatal mortality and morbidity
Source: J Perinatol. 2025 Oct 20;45(12):1801–8. doi: 10.1038/s41372-025-02449-0 (PMC12717003; doi:10.1038/s41372-025-02449-0)
Supplement: Supplementary file 1 — Supplemental Material [file 41372_2025_2449_MOESM1_ESM.docx]

Supplemental Table 1: ICD-9 Codes for Outcome

| Supplemental Table 1. ICD-9-CM Codes for Defined Study Variables | | |
| --- | --- | --- |
| Variables | ICM-9-CM Codes | ICD-10-CM Codes |
| Necrotizing Enterocolitis | 7775, 77750, 77751, 77752, 77753 | P771, P772, P773, P779 |
| Intraventricular Hemorrhage Grade 3 or 4 | 77213, 77214 | P5221, P5222 |
| Chronic Lung Disease | 7707 | P270, P271, P278, P279 |
| Retinopathy of Prematurity | 36220, 36221, 36223, 36224, 36225, 36226, 36227, 36229 | H35101, H35102, H35103, H35109,  H35121, H35122, H35123, H35129,  H35131, H35132, H35133, H35139,  H35141, H35142, H35143, H35149,  H35151, H35152, H35153, H35159,  H35161, H35162, H35163, H35169,  H35171, H35172, H35173, H35179,  H3520, H3521, H3522, H3523 |
| Any hypertension | 64200, 64201, 64202, 64203, 64204, 64210, 64211, 64212, 64213, 64214, 64220, 64221, 64222, 64223, 64224, 64230, 64232, 64233, 64234, 64240, 64241, 64242, 64243, 64244, 64250, 64251, 64252, 64253, 64254, 64270, 64271, 64272, 64273, 64274 | I10, O100, O1001, O1002, O1003, O101, O1011, O1012, O1013, O102, O1021, O1022, O1023, O103, O1031, O1032, O1033, O104, O1041, O1042, O1043, O109, O1091, O1092, O1093  O111, O112, O114, O114, O115, O119  O131, O132, O133, O134, O135, O139 |
| Any diabetes | 6488, 64880, 64882, 64883, 64884, 250, 2500, 25000, 25001, 25002, 25003, 2501, 25010, 25011, 25012, 25013, 2502, 25020, 25021, 25022, 25023, 2503, 25030, 25031, 25032, 25033, 2504, 25040, 25041, 25042, 25043, 2505, 25050, 25051, 25052, 25053, 2506, 25060, 25061, 25062, 25063, 2507, 25070, 25071, 25072, 25073, 2508, 25080, 25081, 25082, 25083, 2509, 25090, 25091, 25092, 25093, 3572, 3602, 36201, 36641, 6480, 64800, 64801, 64802, 64803, 64804 | E080, E081, E082, E083, E084, E085, E086, E088, E089, E090, E091, E092, E093, E094, E095, E096, E098, E099, E101, E102, E103, E104, E105, E106, E108, E109,E110, E111, E112, E113, E114, E115, E116, E118, E119, E12, E130, E131, E132, E133, E134, E135, E136, E138, E139,O240, O241, O243, O244, O248, O249, Z794 |
| Any infection | 0031, 0312, 0362, 0380, 0381, 03810, 03811, 03812, 03819, 0382, 0383, 03840, 03841,  03842, 03843, 03844, 03849, 0388, 0389, 0545, 05472, 77181, 77183, 7907, 99932, 00321, 0360, 0361, 09040, 09042, 09049, 09489, 0949, 3200, 3201, 3202, 3203, 3207, 32081, 32082, 32089, 3209, 3229, 3240, 3241, 3249, 325, 326, 09941, 59000, 59010, 59011, 5902, 59080, 5909, 5950, 59589, 5959, 5990, 77182, 00322, 01180, 01190, 01194, 0310, 0339, 0391, 481, 4820, 4821, 4822, 48230, 48231, 48232, 48239, 48240, 48241, 48242, 48249, 48281, 48282, 48283, 48289, 4829, 4830, 4831, 4838, 4843, 4848, 485, 486, 5130, 0010, 0030, 0040, 0051, 0052, 0053, 00589, 0059, 00800, 00804, 00809, 0082, 00841, 00842, 00843, 00845, 00846, 00847, 00849, 0085, 0090, 0091, 0092, 0392, 11285, 5695, 5582, 5670, 56723, 56729, 5678, 56789, 5679, 01402, 01404, 0022, 0023, 00320, 0038, 0039, 0041, 0048, 0049, 01000, 01890, 0208, 0209, 0239, 024, 0270, 0271, 0300, 0308, 0319, 03289, 0329, 0330, 0331, 0363, 03689, 0369, 037, 0398, 0399, 0400, 0403, 04041, 04082, 04089, 04100, 04101, 04102, 04103, 04104, 04105, 04109, 04110, 04111, 04112, 04119, 0412, 0413, 0414, 04141, 04149, 0415, 0416, 0417, 04181, 04182, 04183, 04184, 04185, 04186, 04189, 0419, 0739, 07888, 07988, 07998, 08881, 0900, 0901, 0902, 0905, 0907, 0909, 0919, 0920, 0929, 09389, 096, 0971, 0979, 0318, 0318, V091, V092, V093, V094, V0950, V0951, V096, V0970, V0980, V0981, V0990, V0991, V021, V022, V023, V0251, V0252, V0253, V0254, V0259, V027, V028, 7718, 77189, 9993, 99931, 99939, V1200, V1201, V1204, V1209, 0761, 0769, 0783, 01500, 01504, 01700, 03281, 03285, 0340, 035, 07798, 0903, 0913, 0955, 0980, 0982, 09840, 09849, 09886, 09889, 0990, 0993, 09950, 09954, 09959, 0998, 0999, 101, 1027, 37601, 38022, 38023, 38200, 3824, 3829, 38300, 3831, 3839, 4210, 42292, 42490, 42491, 42499, 449, 4510, 45111, 45119, 4512, 45181,  45182, 45183, 45184, 45189, 4519, 4572, 460, 4610, 4612, 4618, 4619, 462, 463, 4640, 46400, 46401, 46410, 46411, 46420, 46421, 46430, 46431, 5131, 51901, 5192, 5225, 5283, 56731, 5673, 5721, 5761, 6040, 6800, 6801, 6802, 6803, 6804, 6805, 6806, 6808, 68100, 68101, 68102, 68110, 68111, 6819, 6820, 6821, 6822, 6823, 6824, 6825, 6826, 6827, 6828, 6829, 683, 684, 6850, 6868, 6869, 71100, 71101, 71102, 71104, 71105, 71106, 71107, 71109, 71181, 71195, 72886, 73000, 73001, 73002, 73003, 73004, 73005, 73006, 73007, 73008, 73009, 73012, 73015, 73016, 73018, 73020, 73021, 73022, 73023, 73024, 73025, 73026, 73027, 73028, 73029, 73030, 73032, 73033, 73035, 73036, 73037, 73038, 73039, 73079, 73081, 73085, 73086, 73088, 73089, 73098, 7713, 7714, 7715, 7716, 99933, 2893, 36000, 36001, 36002, 36003, 36019, 36313, 36320, 37040, 37049, 3708, 3709, 37200, 37300, 3735, 37530, 47871, 5400, 5401, 5409, 541, 542, 7280, 72930, 72939, 7294, 37532, 37533, 38010, 38012, 38013, 38201, 13100, 13101, 4650, 1320, 1321, 1329, 0279, 0070, 0072, 0074, 0838, 0839, 0844, 0846, 08882, 1216, 1218, 1229, 1256, 1259, 1269, 1289, 1302, 1308, 1309, 1318, 1319, 1368, 1369, 1330, 1341, 46450, 4658, 4659, V0260, V0261, V0262, V0269, 04511, 07030, 07031, 07032, 07051, 07054, 07059, 07070, 0709, 5731, 5732, 3220, 0470, 0471, 0478, 0479, 048, 0490, 0491, 0498, 0499, 0530, 05319, 0543, 05829, 0621, 48881, 4800, 4801, 4802, 4808, 4809, 4841, 4870, 00861, 00862, 00863, 00864, 00866, 00867, 00869, 0088, 042, 04500, 04591, 0502, 0527, 0528, 0529, 0539, 05479, 0548, 0549, 0569, 0570, 0578, 0579, 05810, 05889, 05919, 0600, 064, 0663, 0701,  07423, 0743, 0748, 075, 0773, 0785, 07889, 0790, 0791, 0792, 0793, 0794, 07950, 07951, 07953, 07959, 0796, 07981, 07983, 07989, 07999, 4660, 4661, 46611, 46619, 4871, 4878, 488, 4880, 48802, 4881, 48811, 48812, 48882, 7710, 7711, 7712, 05320, 05329, 0540, 05410, 05413, 05419, 0542, 05440, 05441, 05442, 05443, 05449, 05473, 0740, 0778, 07799, 0780, 07810, 07811, 07812, 07819, 464, 11281, 11289, 1129, 1124, 3210, 3210, 11283, 1122, 4846, 4847, 7717, 1125, 1140, 1143, 1149, 11509, 1172, 1173, 1175, 1177, 1179, 118, 1363, 1100, 1101, 1102, 1103, 1104, 1105, 1108, 1109, 1110, 1111, 1118, 1119, 1120, 1121, 1123, 11282, 11284, 71168 32301, 32341, 3238, 32381, 3239 | A021, A327, A400, A401, A408, A409, A4101, A4102, A411, A412, A413, A414, A4150, A4151, A4152, A4153, A4159, A4181, A4189, A419, B377, P360, P3610, P3619, P362, P3630, P3639, P364, P365, P368, P369, P369, P372, R6520, R6521, R7881, T80211A, A5040, A5041, A5049, B1009, G000, G002, G003, G008, G009, G01, G02, G038, G039, G042, G060, G061, G062, A523, A5219, A170, N10, N110, N3000, N3080, N3090, N3091, P393, R8271, T83511A, T83518A, J22, A159, A420, J13, J14, J150, J151, J1520, J15211, J15212, J1529, J153, J154, J155, J156, J157, J158, J159, J160, J168, J17, J180, J181, J182, J188, J189, J850, J851, J860, J869, J95851, P231, P232, P233, P234, P235, P236, P238, P239, A5004, A044, A045, A047, A0472, A048, A049, A088, A09, A421, K612, A020, K652, A5145, A028, A029, A219, A230, A2789, A305, A329, A33, A3700, A3790, A4289, A480, A483, A4851, A488, A4901, A4902, A491, A492, A493, A498, A499, A5008, A5009, A501, A502, A5059, A509, A5279, A530, A539, A7740, A78, B950, B951, B952, B953, B954, B955, B9561, B9562, B957, B958, B960, B961, B9620, B9622, B9629, B963, B964, B965, B966, B967, B9689, K3531, V091, V092, V093, B998, B999, P399, Z220, Z221, Z22321, Z22322, Z22330, Z22338, Z2239, Z224, Z228, Z229, A46, A5001, A5002, A5006, A5007, A5031, A5057, A510, A5274, A5277, A5409, A5430, A5431, A549, A568, A57, A671, A672, A740, A749, H00032, H00033, H6001, H6002, H6010, H6011, H6012, H66002, H66012, H6640, H6642, H6690, H6691, H6692, H6693, H7090, J340, J852, J853, J9851, K047, K6812, K6819, K830, L00, L0100, L0101, L0103, L0109, L0201, L0202, L0211, L02211, L02212, L02213, L02214, L02216, L02222, L0231, L0233, L02411, L02412, L02413, L02414, L02415, L02416, L02419, L02423, L02511, L02512, L02519, L02521, L02522, L02611, L02612, L02619, L02621, L02811, L02818, L02821, L0291, L0292, L03011, L03012, L03019, L03031, L03032, L03111, L03112, L03113, L03114, L03115, L03116, L03119, L03211, L03213, L03221, L03311, L03312, L03313, L03314, L03315, L03316, L03317, L03319, L03811, L03818, L0390, L040, L042, L043, L0592, L081, L0882, M726, M8600, M86021, M86051, M86052, M86061, M86062, M8610, M86112, M86121, M86122, M86132, M86151, M86152,  M86172, M8618, M8619, M86621, M86651, M8668, M868X0, M868X1, M868X2, M868X3, M868X5, M868X6, M868X8, M868X9, M869, P381, P389, P390, P391, T847XXA, T8483XA, T8571XA, T85, 30A, T8579XA, G08, H05011, H10021, H10023, H10029, H1030, H1031, H1032, H1033, H44001, H44002, H44003, H44009, H6090, H6091, H6092, H6093, I301, I330, I38, I8001, I8002, I8011, I8012, I80201, I80211, I80233, I80291, I80299, I803, I808, I809, I891, K113, K122, K352, K353, K3532, K3580, K36, M00011, M00012, M00051, M00052, M00061, M00071, M00072, M00822, M00861, M009, M60003, M60051, M60852, M60862, M60872, M6088, M609, M61141, M65161, N4822, N610, I400, J0190, J020, J029, J0390, J040, J0410, J0411, J042, J0430, J0431, J050, J0510, J060, P394, P398, B86, A598, A599, B589, B59, B662, B80, B832, B851, B852, B880, B948, P371, P373, P378, P379, A8030, B179, B181, B182, B1910, B1920, B199, B251, B941, G030, A850, A858, A86, A870, A872, A878, A879, A888, B003, B004, T85738A, B250, J1000, J1001, J1008, J120, J121, J122, J123, J1289, J129, P230, A080, A0811, A0819, A082, A0832, A0839, A084, A925, A928, B007, B0089, B009, B019, B029, B069, B083, B1081, B20, B258, B259, B269, B2709, B3322, B334, B338, B341, B342, B348, B349, B970, B9710, B9711, B9712, B9719, B9729, B974, B9781, B9789, J101, J1089, J111, J201, J205, J206, J208, J209, J210, J211, J218, J219, P350, P351, P352, P353, P358, P359, A880, J09X2, A6002, A601, A630, B001, B0052, B0053, B0059, B078, B079, B081, B084, B085, B088, B09, B300, B303, B309, J00, L130, J069, B3789, B379, B464, B375, B3749, B371, B441, B3781, B376, B447, B4489, B449, B465, B488, B49, P375, B350, B351, B352, B354, B356, B358, B359, B368, B369, B370, B372, B373, B383, B463, G0481, G0490, G053, J22, T80218A, T80219A, T80212A |

| **Supplemental Table 2. Perinatal Characteristics Overall and by NICU Capacity strain Categories** | | | | | |
| --- | --- | --- | --- | --- | --- |
| Characteristics | **Overall** | **Zero Capacity strain** | **Low Capacity strain**  **(**Deciles 1-3) | **Typical Capacity strain**  (Deciles 4-7) | **High Capacity strain**  (Deciles 8-10) |
|  | N (%) | N (%) | N (%) | N (%) | N (%) |
| **Hospital** | N=64,647 | N=15,700 | N=14,409 | N=23,941 | N=10,597 |
| Hospital Ownership** |  |  |  |  |  |
| Government | 13,861 (21) | 6,615 (42) | 1,727 (12) | 1,628 (7) | 3,891 (37) |
| Non-Profit | 32,390 (50) | 7,242 (46) | 7,304 (51) | 13,300 (56) | 4,544 (43) |
| Profit | 18,396 (28) | 1,843 (12) | 5,378 (37) | 9,013 (38) | 2,162 (20) |
| Hospital Urban Influence Code** |  |  |  |  |  |
| Metropolitan | 62,101 (96) | 15,368 (98) | 13,577 (94) | 23,169 (97) | 9,987 (94) |
| Micropolitan | 2,501 (4) | 303 (2) | 832 (6) | 772 (3) | 594 (6) |
| Noncore | 45 (0) | 29 (0.2) | 0 (0) | 0 (0) | 16 (0) |
| NICU Beds** Mean (SD) | 34 (27) | 9 (5) | 46 (24) | 52 (22) | 12 (8) |
| NICU Level of Care** |  |  |  |  |  |
| 2 | 8,036 (12) | 5,547 (35) | 0 (0) | 39 (0) | 2,450 (23) |
| 3 | 43,198 (67) | 10,153 (65) | 10,465 (73) | 14,433 (60) | 8,147 (77) |
| 4 | 13,413 (21) | 0 (0) | 3,944 (27) | 9,469 (40) | 0 (0) |
| Delivery Volume Category** |  |  |  |  |  |
| 10-500 | 356 (1) | 227 (1) | 0 (0) | 0 (0) | 129 (1) |
| 501-1000 | 4,975 (8) | 3,346 (21) | 0 (0) | 127 (1) | 1,502 (14) |
| 1001-2000 | 17,467 (27) | 8,433 (54) | 1,852 (13) | 2,379 (10) | 4,803 (45) |
| >2000 | 41,849 (65) | 3,694 (24) | 12,557 (87) | 21,435 (90) | 4,163 (39) |
| Abbreviations: GA – Gestational Age; GED – General Education Development; NICU – Neonatal Intensive Care Unit;  ** Indicates p < 0.01, * Indicates p <0.05  Rurality is defined using Urban Influence Codes (UICs). UICs 1 or 2 are metropolitan. UICs 3, 5, or 8 are micropolitan. UICs 4, 6, 7, 9, 10, 11 or 12 are noncore. | | | | | |

| **Supplemental Table 3. Perinatal Characteristics Overall and by Delivery Volume** | | | | | |
| --- | --- | --- | --- | --- | --- |
| **Characteristics** | **Overall** | **10-500** | **501-1000** | **1000-2000** | **>2000** |
|  | N (%) | N (%) | N (%) | N (%) | N (%) |
|  | N=64,647 | N=356 | N=4,975 | N=17,467 | N=41,849 |
| **Exposure** |  |  |  |  |  |
| Zero Capacity strain | 15,700 (24) | 227 (64) | 3,346 (67) | 8,433 (48) | 3,694 (9) |
| Low Capacity strain | 14,409 (22) | 0 (0) | 0 (0) | 1,852 (11) | 12,557 (30) |
| Typical Capacity strain | 23,941 (37) | 0 (0) | 127 (3) | 2,379 (14) | 21,435 (51) |
| High Capacity strain | 10,597 (16) | 129 (36) | 1,502 (30) | 4,803 (27) | 4,163 (10) |
| **Outcome** |  |  |  |  |  |
| Composite | 14,633 (23) | 96 (27) | 729 (15) | 2,677 (15) | 11,131 (27) |
| Term | 6,528 (22) | 49 (30) | 388 (13) | 1,464 (15) | 4,627 (28) |
| Preterm | 8,105 (23) | 47 (24) | 341 (17) | 1,213 (16) | 6,504 (26) |
| **Covariates** |  |  |  |  |  |
| NICU Level |  |  |  |  |  |
| 2 | 8,036 (12) | 356 (100) | 2,673 (54) | 5,007 (29) | 0 (0) |
| 3 | 43,198 (67) | 0 (0) | 2,302 (46) | 12,460 (71) | 28,436 (68) |
| 4 | 13,413 (21) | 0 (0) | 0 (0) | 0 (0) | 13,413 (32) |
| Cesarean Section | 35,326 (55) | 188 (53) | 2,935 (59) | 8,445 (48) | 23,758 (57) |
| Diabetes | 9,199 (14) | 31 (9) | 528 (11) | 2,365 (14) | 6,275 (15) |
| Hypertension | 10,672 (17) | 23 (6) | 645 (13) | 2,311 (13) | 7,693 (18) |
| Any Smoking | 13,000 (20) | 123 (35) | 1,107 (22) | 3,292 (19) | 8,478 (20) |
| Gestational Age |  |  |  |  |  |
| 22-27 | 3,433 (5) | 22 (6) | 127 (3) | 476 (3) | 2,808 (7) |
| 28-31 | 6,014 (9) | 33 (9) | 176 (4) | 854 (5) | 4,951 (12) |
| 32-34 | 14,323 (22) | 67 (19) | 797 (16) | 2,795 (16) | 10,664 (25) |
| 35-36 | 11,272 (17) | 70 (20) | 879 (18) | 3,460 (20) | 6,863 (16) |
| 37+ | 29,605 (46) | 164 (46) | 2,996 (60) | 9,882 (57) | 16,563 (40) |
| Female | 29,538 (46) | 153 (43) | 2,243 (45) | 7,957 (46) | 19,185 (46) |
| SGA | 9,611 (15) | 63 (18) | 727 (15) | 2,375 (14) | 6,446 (15) |
| Multiple Gestation | 8,730 (14) | 39 (11) | 401 (8) | 1,751 (10) | 6,539 (16) |
| Congenital Anomaly | 4,218 (7) | 23 (6) | 175 (4) | 603 (3) | 3,417 (8) |
| Abbreviations: GA – Gestational Age; NICU – Neonatal Intensive Care Unit; | | | | | |

Supplemental Figure 1. Consort Flow Diagram


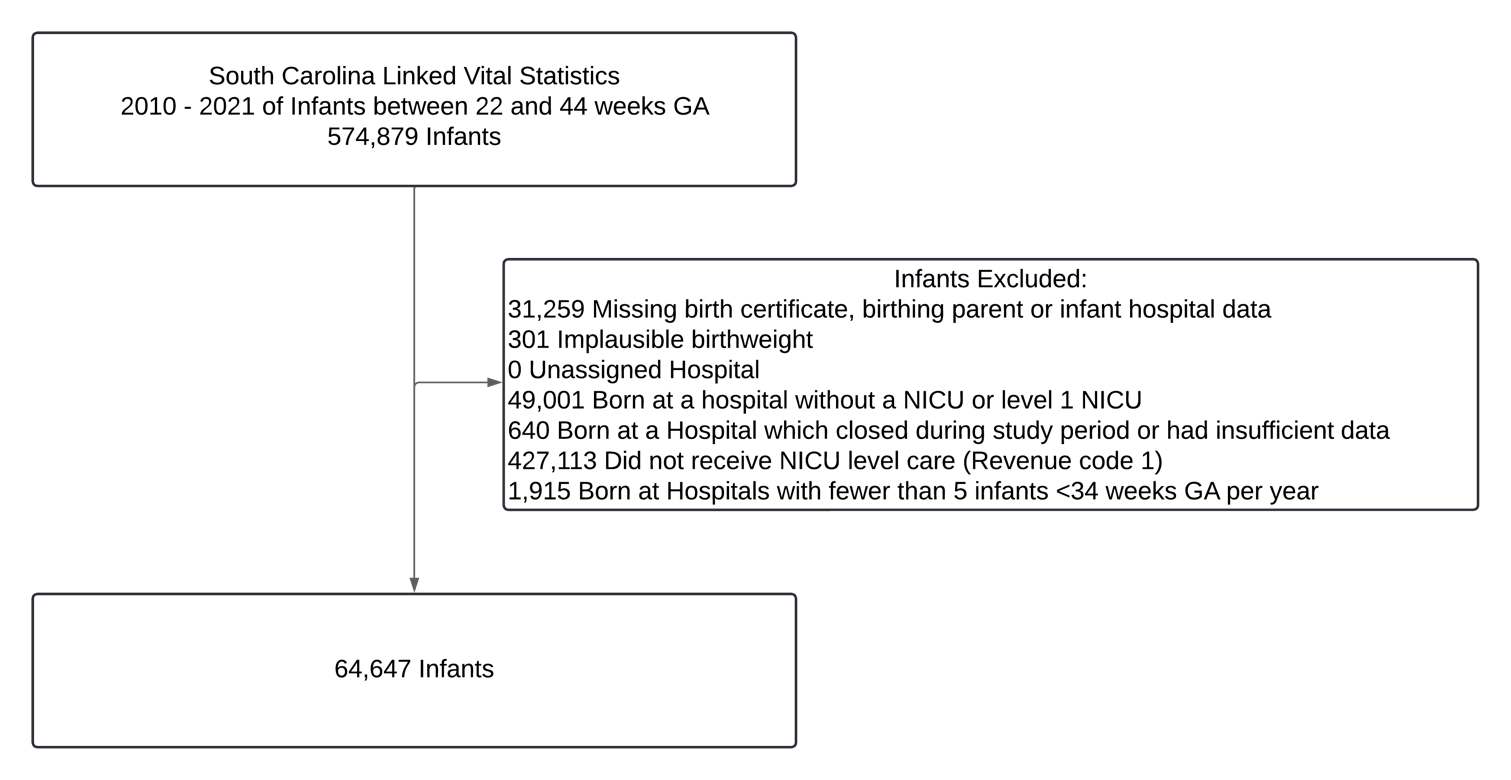


Supplemental Figure 2. NICU Capacity strain and Composite Outcome with Level 2 Units Excluded


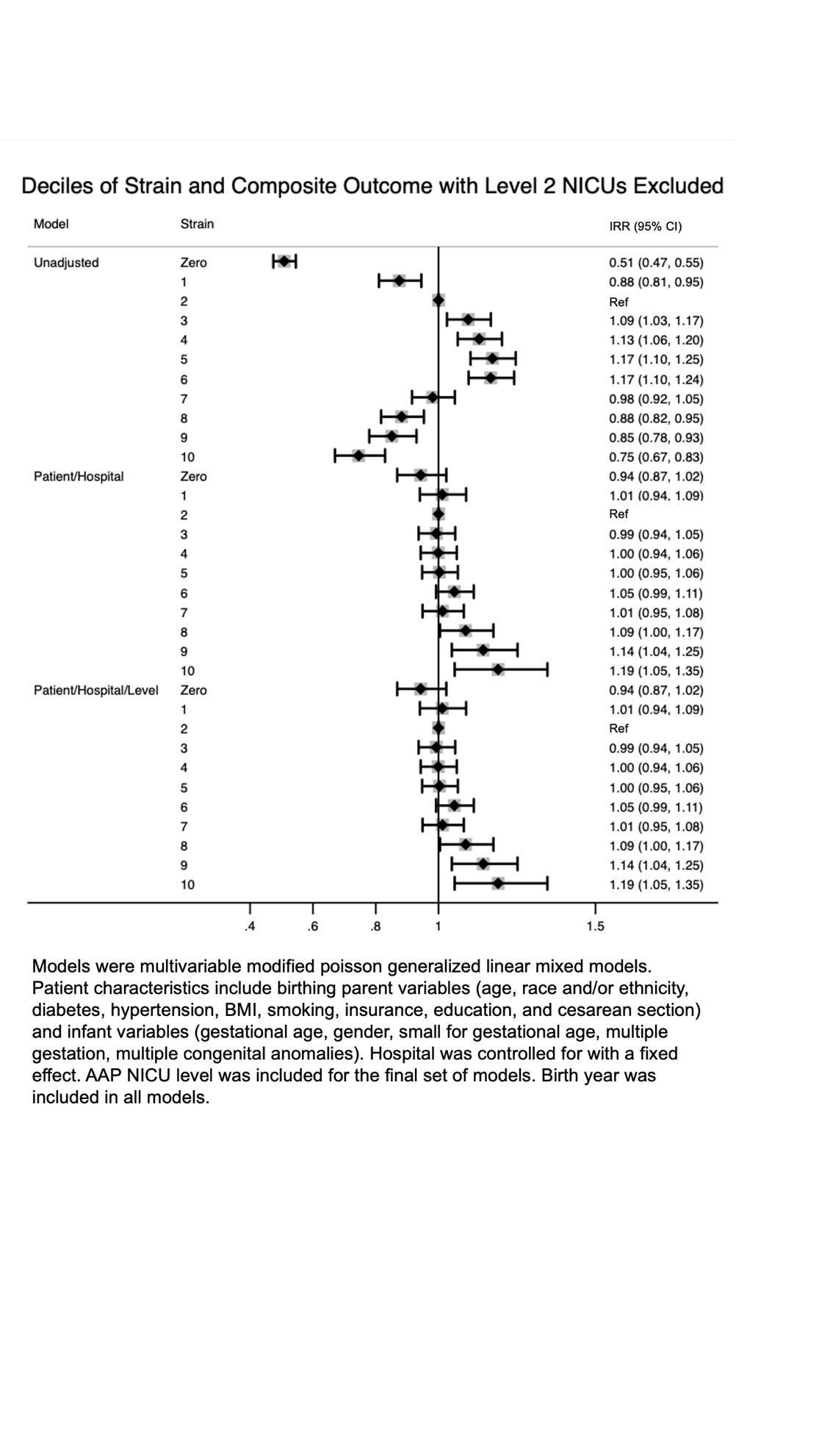


Supplemental Figure 3. NICU Capacity strain and Composite Outcome with Zero Capacity strain Values Excluded


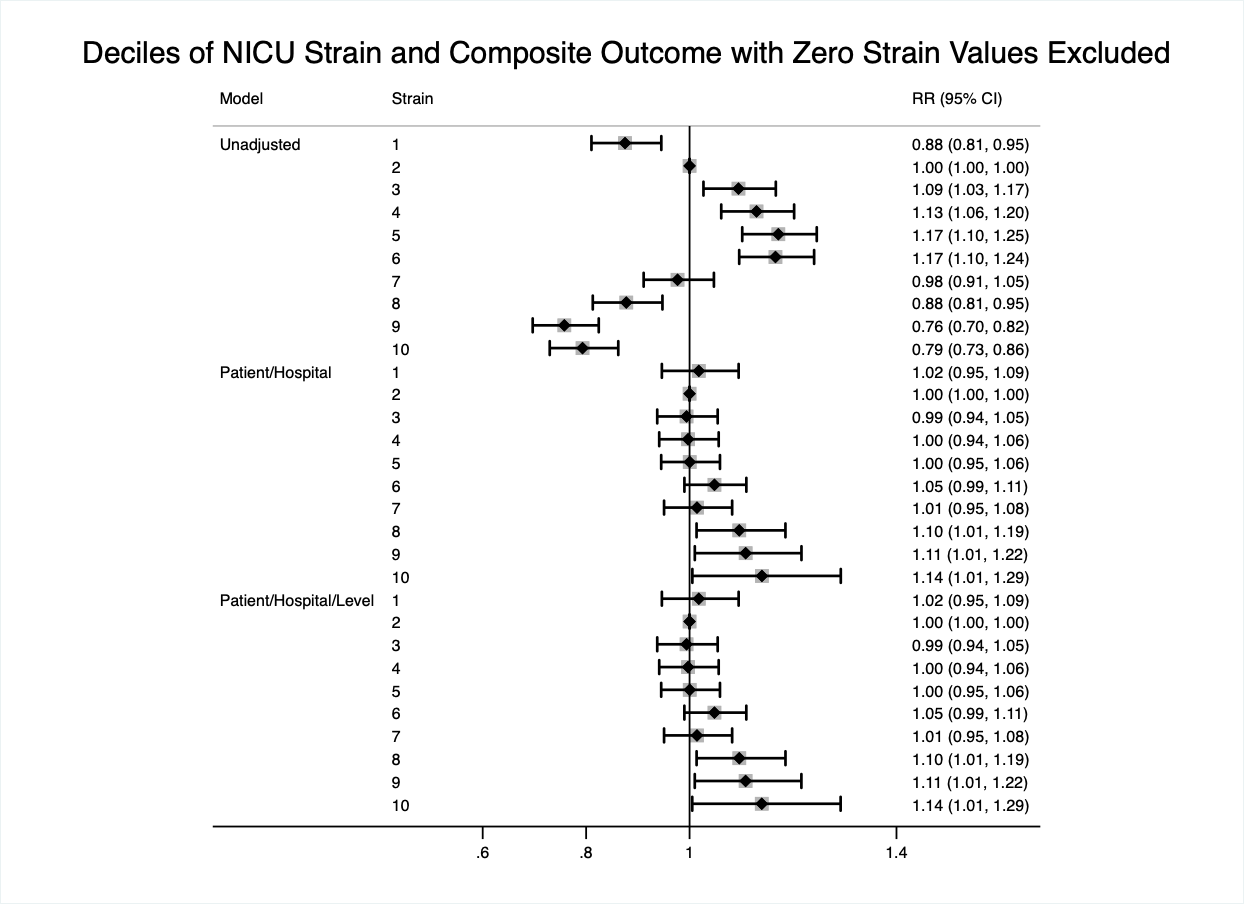


Models were multivariable modified poisson generalized linear mixed models. Patient characteristics include birthing parent variables (age, race and/or ethnicity, diabetes, hypertension, BMI, smoking, insurance, education, and cesarean section) and infant variables (gestational age, gender, small for gestational age, multiple gestation, multiple congenital anomalies). Hospital was controlled for with a fixed effect. AAP NICU level was included for the final set of models. Birth year was included in all models.

Supplemental Figure 4. NICU Admissions and Composite Outcome


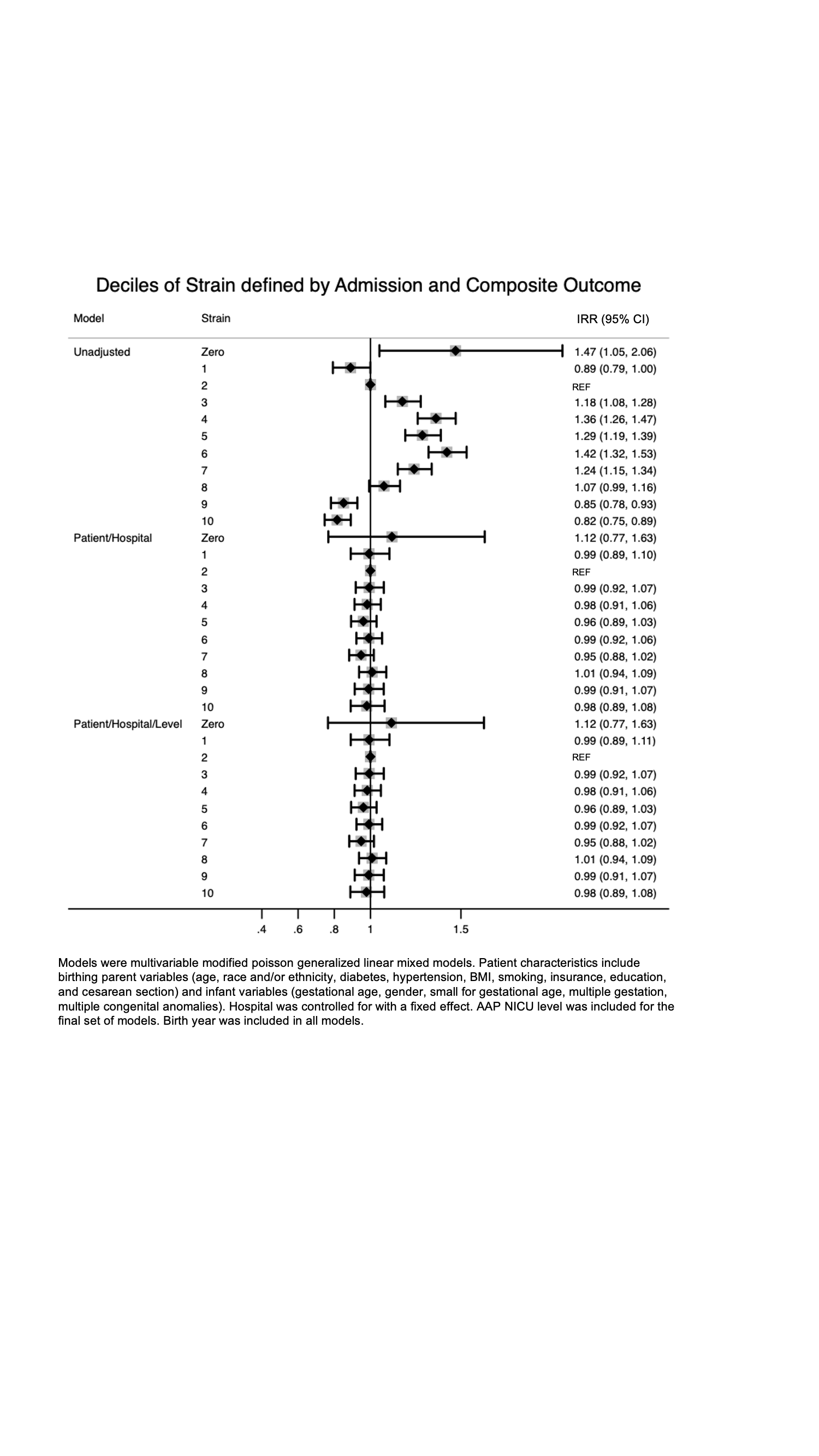


Supplemental Figure 5. NICU Admissions and Term and Preterm Outcomes


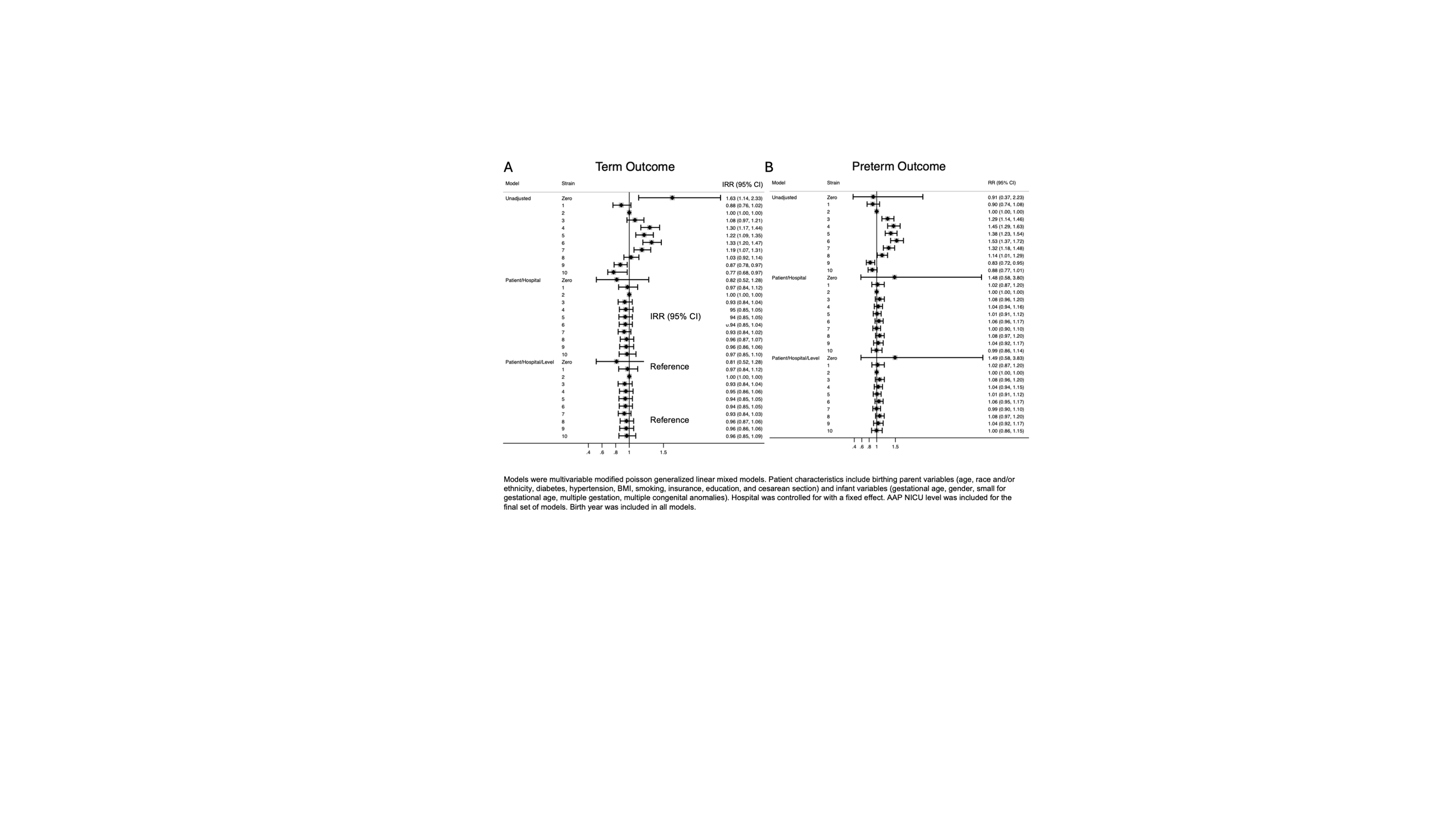


**Supplemental Methods: Sample Capacity strain Calculation**

**NICU A: High Volume**

Consider NICU A with an average annual census of 40 total babies. This NICU has an average annual census of 20 babies <34 weeks. It also has an average annual census of 5 babies with congenital anomalies. For this NICU, the average annual hospital census_<34 weeks GA+Anomalies_ will be 25. Infants both <34 weeks with congenital anomalies are counted once.

High Capacity strain

On January 1, 2024, NICU A has 25 babies <34 weeks admitted as well as 10 babies with congenital anomalies. This would lead to a Daily Census_<34wGA+Anomalies_ of 35 babies.

NICU capacity strain can then be calculated using the following formula:

NICU Capacity strain = [ Daily Census_<34wGA+Anomalies_  - Average annual hospital census_<34 weeks GA+Anomalies_ ] / Average annual hospital census_<34 weeks GA+Anomalies_

NICU Capacity strain = [ 35 – 25 ] / 25 = 10 / 25 = 0.4

Low Volume

On February 1, 2024, NICU A has 10 babies <34 weeks or with congenital anomalies (daily Census_<34wGA+Anomalies_ of 0 babies). Negative values are included in the lowest category.

NICU Capacity strain = [ 10 – 25 ] / 25 = -15 / 25 = -0.4

**NICU B: Low Volume**

Consider NICU B with an average annual census of 10 babies. This NICU cares for primarily term health infants. The NICU has an average annual census of 0.5 babies <34 weeks and with congenital anomalies.

High Capacity strain

On January 1, 2024, NICU B has 2 babies <34 weeks and 1 with congenital anomalies. This would lead to a daily Census_<34wGA+Anomalies_ of 3 babies.

NICU Capacity strain = [ 3 – 0.5 ] / 0.5 = 5

Low Capacity strain

On February 1, 2024, NICU B has no babies <34 weeks or with congenital anomalies (daily Census_<34wGA+Anomalies_ of 0 babies).

NICU Capacity strain = [ 0 – 0.5 ] / 0.5 = 0
